# Both Geography and Ecology Contribute to Mating Isolation in Guppies

**DOI:** 10.1371/journal.pone.0015659

**Published:** 2010-12-15

**Authors:** Amy K. Schwartz, Dylan J. Weese, Paul Bentzen, Michael T. Kinnison, Andrew P. Hendry

**Affiliations:** 1 Redpath Museum and Department of Biology, McGill University, Montreal, Canada; 2 Department of Biological Sciences, University of Maine, Orono, Maine, United States of America; 3 Department of Biology, Dalhousie University, Halifax, Canada; University of Liverpool, United Kingdom

## Abstract

Local adaptation to different environments can promote mating isolation – either as an incidental by-product of trait divergence, or as a result of selection to avoid maladaptive mating. Numerous recent empirical examples point to the common influence of divergent natural selection on speciation based largely on evidence of strong pre-mating isolation between populations from different habitat types. Accumulating evidence for natural selection's influence on speciation is therefore no longer a challenge. The difficulty, rather, is in determining the mechanisms involved in the progress of adaptive divergence to speciation once barriers to gene flow are already present. Here, we present results of both laboratory and field experiments with Trinidadian guppies (*Poecilia reticulata)* from different environments, who do not show complete reproductive isolation despite adaptive divergence. We investigate patterns of mating isolation between populations that do and do not exchange migrants and show evidence for both by-product and reinforcement mechanisms depending on female ecology. Specifically, low-predation females discriminate against all high-predation males thus implying a by-product mechanism, whereas high-predation females only discriminate against low-predation males from further upstream in the same river, implying selection to avoid maladaptive mating. Our study thus confirms that mechanisms of adaptive speciation are not necessarily mutually exclusive and uncovers the complex ecology-geography interactions that underlie the evolution of mating isolation in nature.

## Introduction

Renewed interest in speciation mechanisms has revealed that divergent natural selection can be a powerful promoter of mating isolation, such that stronger pre-zygotic barriers to gene flow evolve between populations from more divergent environments, i.e. “ecological speciation” [1], [2]. When this result holds independent of the geographic relationships among populations, then mating isolation is inferred to be an incidental by-product of adaptive divergence [3]–[6]. In this case, the geographic context is largely irrelevant, as long as the traits under divergent selection also influence mating success. But the geographic context might also be important, with the potential for dispersal influencing the type and degree of mating isolation that evolves. In some cases, dispersal between environments leads to recombination that impedes mating isolation [7]. In other cases, dispersal may strengthen mating isolation as a consequence of selection to avoid maladaptive mating between individuals from different environments [8]. This latter selection can be indirect, through the reduced fitness of hybrid offspring, or direct through costs associated with the act of between-type courtship or mating [9], [10]. These potential positive effects of dispersal on speciation have been suggested in several laboratory studies reporting that mating isolation between ecologically-differentiated populations is stronger when they can exchange migrants in nature [11]–[13].

We used laboratory and field experiments to resolve how interactions between ecology (adaptation to different environments) and geography (potential for dispersal) influence the evolution of mating isolation. When reproductive isolation is complete or nearly complete, it becomes more difficult to determine how barriers to gene flow initially arose - although there is no guarantee that those same barriers will complete it. For this reason, we focus on differentiated populations *within* a species – Trinidadian guppies (*Poecilia reticulata)* - where any reproductive barriers would be those that evolve early in the process of diversification.

The rivers of Trinidad's Northern mountain range offer an excellent system to integrate evolutionary processes (natural and sexual selection) and environmental contexts (ecology and geography) in the study of speciation. In general, predation intensity varies along the upstream-downstream axis, with sharp changes occurring across waterfalls that prevent upstream colonization by predatory fishes [14], [15]. As a result, headwaters and tributaries are generally characterized by low predation, whereas downstream sections and the main channel areas are generally characterized by high predation. Specifically, guppies in downstream sections of rivers on the southern slope of the mountains coexist with the pike cichlid (*Crenicichla alta)* – a large piscivore, whereas upstream guppies coexist with only the killifish (*Rivulus haarti*) – a predator of mild to minimal effect. In contrast, the downstream reaches of rivers on the northern slopes of the mountains, which meet the Carribean sea, were colonized by piscivorous fish of marine origins, such as the mountain mullet *(Agonostomus monticola)* and the goby (*Gobiomorus maculatum*), whereas guppies in upstream sites again coexist with the *R. haarti* and another predator of minimal effect; freshwater prawns (*Macrobrachium sp.)* [16], [17].

Regardless of the particular predator assemblage, high- and low-predation populations within a river show adaptive divergence in many traits, including male colour, behaviour, and life history [16]–[20]. This adaptive divergence has occurred independently in multiple drainages (i.e., parallel evolution), as inferred from patterns of geographical separation and genetic variation [16], [21], [22]. Given this strong adaptive divergence, the theory of ecological speciation would predict that high- and low-predation guppies should show positive assortative mating [1]. Although a majority of studies revealing the effects of predation regime on divergence have done so by transplanting guppies from high to low-predation environments (reviewed in [17]), recent work has shown that low-predation migrants do indeed experience strong viability selection in high-predation environments [23]. Furthermore, females in different predation environments appear to differ in the strength and direction of mating preferences for male size and colour [24]–[26]. Selection against migrants and divergent sexual selection between predation regimes might together form a foundation for mating isolation.

Our laboratory experiment employed no-choice mating trials to test whether females are more likely to mate with males from similar predation environments, and whether any such assortment is influenced by the geographic context of the source populations. This experiment used laboratory-reared guppies from paired high- and low-predation populations in three rivers (Aripo, Quare and Yarra; see [26] for site locations) that represent distinct guppy lineages [22]. Individual males from each of the six populations were paired sequentially with virgin females, from both predation environments in both their native river and one of the two foreign rivers (Table S1). A predominant role for ecology would be indicated if males were consistently preferred by females of their own predation type, regardless of the specific rivers from which the test individuals came. A predominant role for geography would be indicated if males were consistently preferred by females from the same (or different) rivers, irrespective of the predation environments from which the test individuals came.

Although laboratory mating trials are able to detect genetic differences in intrinsic female preferences [27], this might not reflect relative male mating success in nature where females are constantly harassed by males [28], [29] and are mating multiply [30], [31]. Male reproductive success is then influenced by a variety of factors that might complicate interpretations based on female responses to male displays – as in the laboratory trials. These complications include sneaky copulations [32], cryptic female choice [33], sperm competition [34], mate choice copying [35], and daily fluctuations in light environments [36]. We therefore complemented the above laboratory experiments with field enclosure experiments in the Marianne River. Here we mimicked the type of between-population interactions that might occur in nature: low-predation fish dispersing into a high-predation environment (rather than the reverse). We specifically focused on dispersing males because males tend to disperse more than females [37] and variation in mating success is higher for males [30]. Multiple males of the two predation types competed for fertilization with resident females in a natural setting. We then used genetic parentage assignment to test for assortative mating by population.

Two different source populations of low-predation males were chosen to test for differences in geography and phenotype independent of predation environment. One source population was located less than 1 km upstream from the focal high-predation site thus reflecting an interaction that occurs in nature (i.e., “parapatric”). The other was located approximately 3.5 km from the high-predation site: 2 km downstream in the Marianne main stem and then 1.5 km upstream in a separate tributary. Given that guppy dispersal is primarily downstream [38], this represents an interaction that would occur much less frequently in nature (i.e., effectively “allopatric”). Indeed, pair-wise estimates of neutral genetic differentiation indicate that gene flow is likely higher from the upstream low-predation site (Fst_MH-MLP_  = 0.195; Fst_MH-MLA_  = 0.312) [39].

## Results

We employed both field and laboratory experimental designs using multiple paired high- and low- predation populations in order to isolate the roles of ecology, geography, and both inter and intra sexual selection on the progress or limitations to the evolution of reproductive isolation. Rather than exposing one mechanism in particular, we found that the above factors all interact. Specifically, our experiments revealed three main interactions. First, we found differences in patterns of mating isolation between females from the different predation environments. Second, both experiments showed that dispersal is necessary to elicit mating isolation only when migrants are maladapted, and third, that mating isolation appears to be strongest in competitive and social contexts.

**Figure 1:**
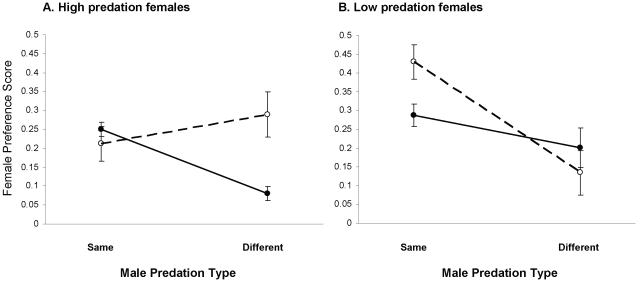
Mean female preference for males related to predation ecology and river. Data points represent least-square means (±s.e) of female preference scores from three rivers within a predation type: (a) high or (b) low. Closed symbols, connected with solid lines, represent preferences for males from the two predation environments in the female's own river; open symbols connected with dashed lines represent allopatric males. See Table 1 for statistical details.

The laboratory study revealed ecological divergence in mate choice as a significant interaction (ANOVA F_3,123_ = 7.34, p = 0.01) between female predation type (high or low) and cross type. We therefore subdivided the data set to determine the nature of this divergence. We first consider low-predation females in the laboratory experiment and then high-predation females in the laboratory and field experiments. Overall, ecology largely determines mating patterns in low-predation females, whereas ecology and geography interact to determine mating patterns in high-predation females (Table 1; Fig.1).

**Table 1:** Variation in female preference as a function of ecological and geographic relationships to test males.

		High Predation Females		Low Predation Females	
	*Factor (fixed)*	*DF* *(num, den)*	*F* *(p -value)*	*DF* *(num, den)*	*F* *(p-value)*
All Males	Male predation(same or different as female)	1, 43.41	0.41(0.53)	1, 44.99	**4.65** **(0.04)**
	Male river(same or different as female)	1, 42.62	1.49(0.23)	1, 43.86	0.005(0.94)
	Male predation x Male river	1, 42.62	**6.81** **(0.01)**	1, 44.02	0.71(0.40)
	Female river(Aripo, Quare, Yarra)	2, 65.3	1.21(0.30)	2, 70.09	0.52(0.6)
	Female river x Male predation	2, 73.19	0.40(0.67)	2, 69.89	0.4(0.67)
	Female river x Male river	2, 74.61	0.45(0.64)	2, 71.22	0.44(0.65)
Parapatric Males Only	Male predation(same or different as female)	1, 43	**8.79** **(0.005)**	1, 43	0.89(0.35)
	Female river(Aripo, Quare, Yarra)	2, 43	0.22(0.80)	2, 43	0.38(0.69)
	Male predation x Female river	2, 43	0.02(0.98)	2, 43	0.47(0.63)
Allopatric Males Only	Male predation(same or different as female)	1, 43	1.73(0.20)	1, 43	**5.49** **(0.02)**
	Female river(Aripo, Quare, Yarra)	2, 43	1.95(0.15)	2, 43	0.46(0.63)
	Male predation x Female river	2, 43	0.67(0.52)	2, 43	0.35(0.71)

Low-predation females in the laboratory experiment discriminated against high-predation males (Table 1; Fig.1), despite the fact that these females evolved in a situation where they almost never encounter such males. This mating isolation was present regardless of whether the high-predation males were from the same or different rivers. Ecology therefore largely overwhelms geography in determining the mating preferences of low-predation females. This is not to say, however, that geography is totally irrelevant. That is, low-predation females were less discriminating against high-predation males from the same river (index of mating isolation, IMI  = 0.233) than they were against high-predation males from a different river (IMI  = 0.413; Table 1; Fig. 1; Table S3).

**Figure 2:**
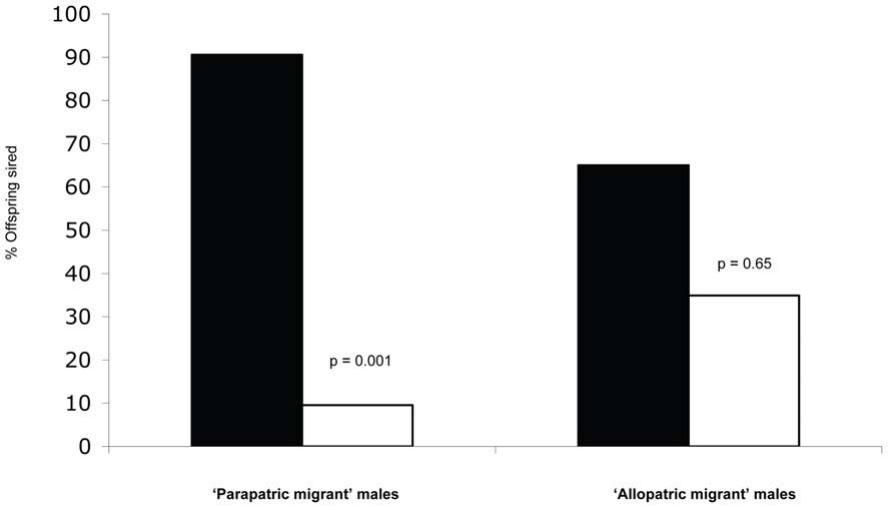
Relative mating success of high- and low-predation males in the wild. Results are shown for two field enclosures in which local, high-predation males (solid bars) competed against low-predation males (open bars) from an upstream population (“parapatric”) and a more distant, downstream population (“allopatric”) for matings with high-predation females. Mating success is shown as the proportion of total offspring sired by males of each population.

High-predation females in the laboratory experiment discriminated against low-predation males from the same river (IMI  = 0.395), but not against low-predation males from different rivers (IMI  = −0.101; Table 1; Fig. 1; Table S3). The same pattern was found in the field experiment (Fig. 2): the relative mating success (proportion of offspring sired) of low-predation males with high-predation females depended on geographic relationships between the populations (χ^2^_1,57_  = 15.32, p<0.0001). On the one hand, high-predation males outcompeted parapatric low-predation males for successful fertilizations with resident females, siring 90.54% of the offspring (χ^2^_1, 45_  = 10.48, p = 0.001). On the other hand, high-predation males sired only 65% of the offspring when in competition with allopatric low-predation males, which did not deviate from random mating (χ^2^_1,24_ = 0.2, p = 0.65).

**Figure 3:**
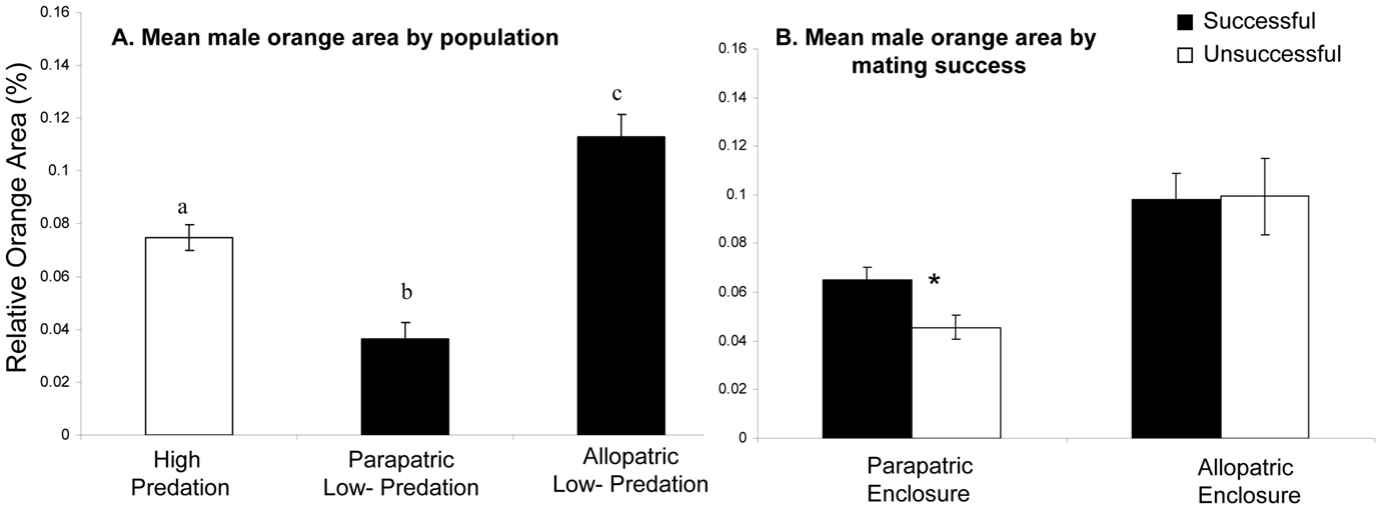
Male colour variation with respect to population and mating success in the field experiment. Mean (+/− s.e) percent of body covered in orange area among males in the three populations (A), and the influence of orange area on mating success (B) in the two enclosures. Mating success here refers to whether or not a male sired at least one offspring.

To further investigate the basis of the observed patterns of mating isolation, we examined the influence of individual male phenotype on his mating success. If mating isolation were evolving as a consequence of divergence in sexually selected traits, then males more phenotypically divergent from the female's native population should be discriminated against most strongly, regardless of predation type [5]. The correlation between the extent of divergence in male trait values and relative male mating success varied depending on context. Overall in the field experiment, males with relatively more orange (a trait subject to female mate choice in a number of populations – see [17], [27] for reviews) were more likely to sire offspring (χ^2^_1, 68_  = 7.48, p = 0.0062). This effect was not consistent between enclosures however, as indicated by a significant enclosure by orange interaction (χ^2^_1, 68_ = 3.97, p = 0.04). The nature of this interaction is illustrated in Figure 3. Specifically, in the ‘parapatric’ enclosure, where high-predation males are on average more orange than low-predation males (ANOVA F_1, 25_ = 30.51, p<0.001) and sired the majority of the offspring, male mating success was positively related to orange colour (χ^2^_1,45_  = 6.76, p = 0.009). In contrast, although males from the ‘allopatric’ low-predation population are substantially more orange than high-predation males (ANOVA F_1,44_  = 9.32, p = 0.004; Table S2), variation in orange here did not influence the likelihood of male mating success (χ^2^_1,24_  = 0.0033, p = 0.95). Finally, in the laboratory trials, there as no indication that variation in orange among males generally influenced the observed patterns of mate choice (High predation females: F_1,81_  = 2.4, p = 0.12; Low-predation females: F_1,95_  = 0.76, p = 0.4).

## Discussion

Our results reinforce the importance of examining interactions between ecological and geographical factors in the early stages of divergence and speciation [1], [2], [40]. Previous studies of mating isolation between predation environments in fish have tended to use only allopatric [6] or parapatric [41] populations. By considering both geographic contexts in the same experiments, we were able to show that the evolution of mating isolation in response to predation intensity depends on the predation ecology of the female population and whether or not the populations interact in nature.

On the one hand, low-predation females routinely discriminated against high-predation males from the same or different rivers (possible by-product evolution), and this was strongest when they were from a different river. Given that low-predation populations were likely originally colonized from high-predation populations in the same river [22], this result suggests that some vestiges of ancestral preferences may persist for a considerable time following the colonization of new environments. This provides support for suggestions that mating preferences might sometimes evolve very slowly, even after environmental change [42]. For example, parapatric high-predation males may experience less mating isolation because they are more similar to upstream low-predation males in certain traits than are allopatric high-predation males. Alternatively, this relatively increased preference for foreign low-predation males may also indicate a general response toward novel or rare phenotypes [43], particularly if preferences are based on traits that exhibit differences across rivers. Within rivers, sexual selection for orange colour is generally stronger in low- than in high-predation populations [24], [26], [44], [45], however there is substantially more variation in all colour traits among low-predation males from different rivers than high-predation males [20], [25], [26]. One likely explanation for this apparent paradox is that in high-predation, where natural selection is stronger on colour [19] (but see [46]), the potential for sexual selection to affect elaboration of male colour is limited. Once viability selection on colour is relaxed however, sexual selection can evolve in arbitrary directions in different populations [5]. The factors that influence polymorphism in guppy colour within and between populations remain a question of active investigation [17], [47], however further investigation in spatial and temporal variation in sexual selection may help to explain the relatively higher variation in both mating preferences and mate signals among low-predation populations. Despite evidence for a by-product mechanism of mating isolation against high-predation males by low-predation females, these results suggest that even when natural and sexual selection target the same trait, sexual selection within populations and sexual isolation between them do not necessarily follow from each other.

High-predation females, on the other hand, only discriminated against low-predation males when they were from the same river in both field and laboratory experiments. For these females in a high-risk environment, mating isolation does not appear to be a by-product of general adaptation to an environment of increased mortality, but rather due to selection against the regular influx of potentially maladapted migrant males or their resulting ‘hybrid’ offspring (i.e. reinforcement).

Although high-predation females consistently show a reduced response to upstream low-predation males and indices of mating isolation for these pairings are the highest observed, the magnitude of this effect is relatively low compared with similar studies [3], [6]. For example, we found a 56.58% difference in female responses to native males, whereas Langerhans et al.[6] report a 251% difference in mating response when a female was given a choice between a male from her own and another predation regime. This relatively lower effect may be due to differences among females in choosiness (i.e. how discriminating a female is) and/or responsiveness (i.e. how much she is willing to mate generally) [48]. While differences in intrinsic female preferences may not be sufficient to effectively reduce gene flow between populations on their own, mating isolation is substantially more pronounced in the field experiment, where males were in direct competition. This suggests that the social environment and mechanisms of intra-sexual selection may be relatively more important factors contributing to sexual selection against migrants than mating preferences themselves [49]–[51].

Furthermore, mating preferences for particular trait values do not appear to predict patterns of mating isolation in high-predation females, which provides further support to rejection the by-product hypothesis in favour of reinforcement in high-predation. Male orange colour is potentially a main target of both mate choice [27] and predation [19], therefore presenting a classic scenario for adaptive speciation. Although we show evidence for patterns of mate discrimination by ecotype (predation), orange colour is not consistently divergent between predation environments, nor does it appear to influence the observed patterns of mate discrimination. Although it initially appears that females prefer males with relatively more orange in the field experiment, this result only holds for the parapatric enclosure where high-predation males are more orange than low-predation males (Figure 3; Table S2), suggesting that it is not phenotypic differences among males or between ecotypes that generally influence differences in relative mating success. The specific traits or trait combinations involved in within and between population mate choice and mating success therefore remain to be determined. Nonetheless, the interactions between ecology, geography and social context on mating isolation detected here highlight the importance of expanding the predictions of speciation models to incorporate detailed investigations of the phylogenetic history, current ecological differences, and the level of dispersal between populations.

## Materials and Methods

### Ethics Statement

All experiments and housing conditions of animals were conducted with the approval of the Canadian Council for Animal Care (CCAC) and McGill University's Animal Care Committee (Protocol # 4570). Import and export permits of guppies were graciously provided by the Department of Fisheries and Agriculture, Trinidad, W.I.

### Laboratory experiments

All experiments used the offspring of females collected from six populations along Trinidad's Northern Range mountains. These populations were paired upstream (low-predation) and downstream (high-predation) localities in three separate rivers (Aripo, Quare, Yarra; Table S1). As juveniles, these offspring were raised together in family-specific tanks and males were removed upon maturity and reared separately with untested females from their own population. Lab-born females therefore remained virgins, a state in which they are more receptive to mating [52].

Mate choice trials involved placing single pairs of males and females into aquaria and recording their behaviour for thirty minutes or until copulation, whichever came first. Female preference for a given male was then scored from video following standard protocols [26], [27], [45], [50]–[53], in which standardized female responses to male displays yield a “fractional intensity of response” (FIR). Briefly, FIR is achieved by calculating the sum of the intensity of each female's typical glide response on a scale of 0–5 (with 5 indicating successful copulation, and 0 ignoring the male [27]) to each male display which is then standardized for the maximum response possible within a given trial [26].

Although 36 possible pair-wise combinations were possible between the six populations, this was not logistically feasible. We therefore followed previous related studies of ecological speciation [3], [5] by performing a series of cross types that used a subset of the populations: *(1)*same predation and same river, *(2)* same predation and different river, *(3)* different predation and same river, and *(4)* different predation and different river (Table S1). Each male was tested sequentially with a different female at intervals of 24 hours, with each test corresponding to one of the four cross types. Sequential testing of different females with each male allowed us to statistically control for male variation within populations. The order of these female types was randomized for each male; but, regardless, trial order did not affect male courtship behaviour (e.g., sneaking rate: repeated measures ANOVA, F_1,57_  = 0.2, p = 0.90; display rate: F_1,57_ = 0.19, p = 0.89).

Variation in female preference (FIR) was analyzed with generalized linear mixed models (GLMM) that included male identity as a random factor. Fixed factors included female predation type (high or low), female river of origin (Aripo, Quare, Yarra), male river cross type (same or different with respect to the female), male predation cross type (same or different with respect to the female), and all possible interactions. By-product mating isolation would be indicated by a significant effect of male predation cross type that was consistent across all rivers; i.e., with a non-significant interaction with female river. Significance was assessed using restricted maximum likelihood as implemented in SAS.

Male body and colour spot size were measured from digital photographs using standard protocols [19], [20] with Image J image software (http://rsbweb.nih.gov/ij/). The influence of male orange colouration on relative mating success was examined in two ways. First, male trait values were included as a factor in the GLMM above. Second, linear regressions were used within each female population to assess the relationship between female FIR for a given male and the difference between his phenotype from the average trait value in the female's population. A negative correlation would support the hypothesis that mating isolation is driven by divergent selection on male colour since foreign males with phenotypes closest to a female's local resident males would have the highest mating success.

An index of mating isolation (IMI) was calculated as the difference between the average preference (FIR) of females from a given population for their local males relative to foreign males of a given cross type (FIR own – FIR foreign/FIR own + FIR foreign) [6]. Three types of indices where calculated based on the predation contrast and geographic context of the foreign male: different predation type from the same river, different predation type from a different river, and same predation type from a different river. This index ranges from -1 to +1 with 0 indicating no mating isolation and a positive value indicating preference for the native male.

### Field-enclosure experiment

Juveniles were collected from a high-predation section of the Marianne River (MH) and raised to maturity in the laboratory with the sexes kept separate. Forty-six of the resulting virgin females were split randomly into two experimental enclosures in their home environment (25 females in Enclosure A and 21 females in Enclosure B). Enclosures were constructed by isolating side-channel habitats with chicken wire and mesh fabric, therefore excluding both local guppies and aquatic predators but allowing for otherwise natural habitat conditions and water flow. Local high-predation males (MH) and foreign low-predation males (MLA or MLP- see Table S2) were collected from the wild and held in the laboratory for one day during which time they were photographed and had scales removed for DNA analysis. They were then released into the enclosures that already contained females. After 48 hours in the enclosures, females were captured and returned to the laboratory where they were isolated for three weeks. Females were then killed with an overdose of MS-222 and four offspring were dissected from each.

Paternity was determined based on allele-sharing at six tetra-nucleotide microsatellite markers: Pre8, Pre9, Pre15, Pre46, Pre32, and Pre53 [54], [55]. DNA was extracted as previously described [54] from either scale samples (candidate parents) or whole tissue (embryos). Individual assignment of offspring to potential fathers in their enclosure was conducted with a likelihood-based exclusion analysis in the program CERVUS 3.0 [56], [57] based on the difference in log-likelihood scores between candidate fathers. The power of assignment based on these markers was relatively low at the individual level but was very high at the source population level. Specifically, 93.67% of the offspring in Enclosure A and 88.3% of the offspring in Enclosure B could be unambiguously assigned to either local high-predation (MH) or foreign low-predation (MLA or MLP) males. Relative mating success of the two male ecotypes within each enclosure was statistically analyzed with contingency analysis. For those offspring that assigned to individual males with 95% confidence, we then used logistic regression with enclosure replicate as a fixed factor to determine if individual male mating success was associated with colour.

## Supporting Information

Table S1 Details of crosses performed in the laboratory no-choice mating experiment. Note that each male was tested sequentially with four different females, one of each cross type, whereas females were tested only once.
(DOC)

Table S2 Geographic information of site locations and sample sizes for populations used in the field enclosure experiment. Grid references are from the Trinidad National Grid System 1∶25,000 map series. Also shown are least-square means (+/- standard errors) and results of analysis of variance in male relative orange area. Superscripts indicate homogeneous subsets from post-hoc Tukey tests examining variation among populations.
(DOC)

Table S3 Indices of ecological and geographical mating isolation by female population. Because three types of foreign crosses were performed (see text), multiple indices can be calculated. The three shown here inform different hypotheses about the roles of ecology, geography and their interaction in mating isolation. “Parapatric ecological mating isolation” compares female preferences for a local male to a male from the same river but different predation type; “allopatric ecological mating isolation” compares local preferences to preferences for males from a different river and predation type; “geographic mating isolation” compares local preferences to preferences for males from a different river but the same predation type.
(DOC)
