# Supplementary material for: Both Geography and Ecology Contribute to Mating Isolation in Guppies
Source: PLoS One. 2010 Dec 15;5(12):e15659. doi: 10.1371/journal.pone.0015659 (PMC3002288; doi:10.1371/journal.pone.0015659)
Supplement: Table S1 — Details of crosses performed in the laboratory no-choice mating experiment. Note that each male was tested sequentially with four different females, one of each cross type, whereas females were tested only once. (DOC) [file pone.0015659.s001.doc]

Table S1.

|  | *Cross type (female population)* | | | |
| --- | --- | --- | --- | --- |
|  | Same Predation,  Same River | Same Predation,  Different River | Different Predation,  Same River | Different Predation, Different River |
| *Male Population* |  |  |  |  |
| Aripo Low | Aripo Low | Quare Low | Aripo High | Quare High |
| Aripo High | Aripo High | Quare High | Aripo Low | Quare Low |
| Quare Low | Quare Low | Yarra Low | Quare High | Yarra High |
| Quare High | Quare High | Yarra High | Quare Low | Yarra Low |
| Yarra Low | Yarra Low | Aripo Low | Yarra High | Aripo High |
| Yarra High | Yarra High | Aripo Low | Yarra Low | Aripo Low |
