# Supplementary material for: Both Geography and Ecology Contribute to Mating Isolation in Guppies
Source: PLoS One. 2010 Dec 15;5(12):e15659. doi: 10.1371/journal.pone.0015659 (PMC3002288; doi:10.1371/journal.pone.0015659)
Supplement: Table S2 — Geographic information of site locations and sample sizes for populations used in the field enclosure experiment. Grid references are from the Trinidad National Grid System 1∶25,000 map series. Also shown are least-square means (+/- standard errors) and results of analysis of variance in male relative orange area. Superscripts indicate homogeneous subsets from post-hoc Tukey tests examining variation among populations. (DOC) [file pone.0015659.s002.doc]

Table S2.

| Population | Predation | N  (males,females) | Drainage | Grid Reference | Mean relative orange area (%) |
| --- | --- | --- | --- | --- | --- |
| *Field Experiment* |  |  |  |  |  |
| Marianne ‘MH’ | High | 35, 46 | Northern | 280 E 399 N | 7.45 (0.49)^a^ |
| Marianne  ‘MLP’ | Low | 22, n/a | Northern | 189 E 338N | 3.65 (0.62)^b^ |
| Marianne  ‘MLA’ | Low | 12, n/a | Northern | 720 E 358 N | 11.31 (0.84)^c^ |
| F-statistic |  |  |  |  | 27.81 |
| p-value |  |  |  |  | <0.0001 |
