# Supplementary material for: Both Geography and Ecology Contribute to Mating Isolation in Guppies
Source: PLoS One. 2010 Dec 15;5(12):e15659. doi: 10.1371/journal.pone.0015659 (PMC3002288; doi:10.1371/journal.pone.0015659)
Supplement: Table S3 — Indices of ecological and geographical mating isolation by female population. Because three types of foreign crosses were performed (see text), multiple indices can be calculated. The three shown here inform different hypotheses about the roles of ecology, geography and their interaction in mating isolation. “Parapatric ecological mating isolation” compares female preferences for a local male to a male from the same river but different predation type; “allopatric ecological mating isolation” compares local preferences to preferences for males from a different river and predation type; “geographic mating isolation” compares local preferences to preferences for males from a different river but the same predation type. (DOC) [file pone.0015659.s003.doc]

Table S3.

| Female Population | | Parapatric Ecological Mating Isolation  (± s.e.) | Allopatric Ecological Mating Isolation  (± s.e.) | Geographic Mating Isolation (± s.e.) |
| --- | --- | --- | --- | --- |
| River | Predation |  |  |  |
| Aripo | High | 0.405 (0.070) | -0.443 (0.189) | 0.386 (0.082) |
| Quare | High | 0.348 (0.131) | -0.026 (0.082) | -0.116 (0.138) |
| Yarra | High | 0.433 (0.144) | 0.167 (0.179) | 0.101 (0.133) |
| All | High | 0.395 (0.059) | -0.101 (0.062) | 0.124 (0.061) |
| Aripo | Low | 0.133 (0.156) | 0.307 (0.183) | -0.307 (0.197) |
| Quare | Low | 0.343 (0.164) | 0.447 (0.190) | -0.566 (0.130) |
| Yarra | Low | 0.224 (0.262) | 0.486 (0.052) | 0.381 (0.045) |
| All | Low | 0.233 (0.056) | 0.413 (0.055) | -0.164 (0.062) |
